# Reprogramming of glucose metabolism: Metabolic alterations in the progression of osteosarcoma

**DOI:** 10.1016/j.jbo.2024.100521

**Published:** 2024-01-04

**Authors:** Fangyu An, Weirong Chang, Jiayi Song, Jie Zhang, Zhonghong Li, Peng Gao, Yujie Wang, Zhipan Xiao, Chunlu Yan

**Affiliations:** a Teaching Experiment Training Center, Gansu University of Chinese Medicine, Lanzhou 730000, Gansu, China; b School of Basic Medicine, Gansu University of Chinese Medicine, Lanzhou 730000, Gansu, China; c School of Tradional Chinese and Werstern Medicine, Gansu University of Chinese Medicine, Lanzhou 730000, Gansu, China

**Keywords:** Osteosarcoma, Glucose metabolism, Key enzymes, Signaling pathways, Targeted therapy

## Abstract

Metabolic reprogramming is an adaptive response of tumour cells under hypoxia and low nutrition conditions. There is increasing evidence that glucose metabolism reprogramming can regulate the growth and metastasis of osteosarcoma (OS). Reprogramming in the progress of OS can bring opportunities for early diagnosis and treatment of OS. Previous research mainly focused on the glycolytic pathway of glucose metabolism, often neglecting the tricarboxylic acid cycle and pentose phosphate pathway. However, the tricarboxylic acid cycle and pentose phosphate pathway of glucose metabolism are also involved in the progression of OS and are closely related to this disease. The research on glucose metabolism in OS has not yet been summarized. In this review, we discuss the abnormal expression of key molecules related to glucose metabolism in OS and summarize the glucose metabolism related signaling pathways involved in the occurrence and development of OS. In addition, we discuss some of the targeted drugs that regulate glucose metabolism pathways, which can lead to effective strategies for targeted treatment of OS.

## Highlights

• My manuscript discusses the importance of metabolic reprogramming, particularly in glucose metabolism, as an adaptive response of tumor cells under hypoxia and low nutrition conditions. It highlights the increasing evidence that glucose metabolism reprogramming plays a role in regulating the growth and metastasis of osteosarcoma (OS). The manuscript points out that previous research has primarily focused on the glycolytic pathway of glucose metabolism, neglecting the tricarboxylic acid cycle and pentose phosphate pathway, despite their involvement in OS progression. The authors emphasize the need to summarize the research on glucose metabolism in OS. They review the abnormal expression of key molecules related to sugar metabolism and summarize the sugar metabolism-related signaling pathways involved in the occurrence and development of OS. Additionally, the manuscript discusses targeted drugs that regulate sugar metabolism pathways and their potential for effective targeted treatment of OS.
• My manuscript addresses an important topic in cancer research, specifically the role of metabolic reprogramming and glucose metabolism in osteosarcoma. The authors highlight the existing gap in understanding the involvement of the tricarboxylic acid cycle and pentose phosphate pathway in OS, which suggests the need for further investigation in this area. The review of abnormal expression of key molecules and signaling pathways related to sugar metabolism provides valuable insights into the molecular mechanisms underlying OS development. The discussion on targeted drugs that regulate sugar metabolism pathways also opens up possibilities for potential therapeutic strategies. Overall, this manuscript contributes to the field by summarizing and emphasizing the significance of glucose metabolism pathways in osteosarcoma, providing a foundation for future research and potential advancements in diagnosis and treatment.

## 1 Introduction

Osteosarcoma (OS) is the most common primary malignant sarcoma in clinical practice at present. The age of onset is bimodally distributed: the highest incidence rate is among children and adolescents, followed by the elderly (over 60 years old) [1]. Os often occurs in the metaphysis of the long bones of the limbs: femur (42 %; 75 % distal femur), tibia (19 %; 80 % proximal tibia), and humerus (10 %; 90 % proximal humerus). In addition, OS may also occur in the skull or chin (8 %) and pelvis (8 %) [2]. The typical signs and symptoms of OS are local pain, accompanied by local swelling and limited joint movement [3]. At present, the diagnosis of OS mainly relies on the combination of clinical signs, medical history, radiology, blood work, and bone biopsy [4]. However, an effective diagnostic method is lacking. In addition, patients with OS are prone to metastasis, which results in a very low survival rate. Studies have shown that the five-year survival rate of patients with localized OS is approximately 60 %—but only 20 % in patients with metastatic or recurrent diseases [5]. The main reason for this is the lack of effective treatment methods. The current focus in clinical practice is on how to improve the level of diagnosis and treatment, prolong the survival rate of patients, and screen effective targeted drugs.

Material metabolism is a fundamental characteristic of life. The chemical processes related to physiology, such as the digestion, absorption, function, and decomposition of substances in the body, are collectively called material metabolism. At present, many studies have found that regulating enzymes related to glucose metabolism can not only inhibit the growth and metastasis of OS but also reduce the chemotherapy resistance of OS patients [6], [7], bringing new hope to OS patients. Therefore, exploring the changes in enzyme activity related to glucose metabolism and the specific mechanisms of glucose metabolism pathways in OS is of great significance. Metabolomics can provide an overall assessment of cell state from the perspectives of gene regulation, enzyme dynamics changes, and metabolic response changes. Metabolic reprogramming is an adaptive response of tumour cells under hypoxia and low nutrition conditions. Studies have confirmed that glucose metabolism reprogramming is a key factor in the progression of many cancers [8]. However, the mechanism of glucose metabolism in the occurrence and development of OS is largely unknown. Therefore, in this article, we summarize the research progress of glucose metabolism reprogramming in OS, with the aim of providing a basis for the formulation of new treatment strategies for OS.

## 2 Roles of glycometabolism in OS

At present, it is known that glucose metabolism mainly includes three pathways: glycolysis, tricarboxylic acid cycle, and pentose phosphate pathway. Each of these pathways is discussed here in detail.

### 2.1 Glycolysis

The glycolytic pathway is the main source of energy required for the growth and proliferation of OS tumour cells. One molecule of glucose can cleave into two molecules of pyruvate in the cytoplasm and produce two molecules of ATP. There are two main pathways for the production of pyruvate to go, one is to reduce to lactate, and the other is to enter the mitochondria and participate in the tricarboxylic acid cycle. It has been reported that, compared with normal cells, the extracellular acidification rate (ECAR) of OS cells increased and the cell oxygen consumption rate (OCR) decreased [9]. The authors of the study further explored the changes in the structure and function of mitochondria in OS cells and uncovered swelling, depolarization, and loss of membrane integrity of mitochondria in OS cells [9]. This finding indicates that the glycolytic pathway in OS cells is activated, while tricarboxylic acid cycle (TCA) and mitochondrial function is inhibited, indicating the existence of the Warburg effect. Other studies have further confirmed that there is, indeed, a correlation between cancer cells and the Warburg effect [10]. Even under sufficient oxygen supply, cancer cells can still activate the glycolytic pathway while inhibiting the tricarboxylic acid cycle [10]. It can be seen that the glycolytic pathway is involved in the progression of OS. It has also been shown that enhanced aerobic glycolysis can promote the tumorgenic activity of OS cells, and is associated with poor prognosis in patients with OS [11]. In summary, enhanced aerobic glycolysis is the main metabolic change of OS, and Warbug effect may be the key mechanism of enhanced aerobic glycolysis in the progression of OS.

### 2.2 Tricarboxylic acid cycle

Also known as citric acid cycle, the TCA cycle is a circulatory reaction system composed of a series of enzyme catalysis in mitochondria and serves as a hub for the metabolism of glucose, lipid, and amino acids. The first intermediate product is citric acid containing three carboxyl groups, from which it got its name. Because this process was proposed by Krebs, it is also known as the Krebs cycle. The main characteristic of the TCA cycle is the decomposition of acetyl CoA through the tricarboxylic acid cycle, which undergoes a total of eight reactions, mainly involving four dehydrogenations, two decarboxylations, and one substrate level phosphorylation. In tumour cells, most pyruvate is reduced to lactic acid in the cytoplasm, and only a small part of pyruvate enters the mitochondria, where oxidative decarboxylation results in the conversion of pyruvate to acetyl CoA. Mutation/activity loss of isocitric acid dehydrogenase (IDH), succinate dehydrogenase (SDH), and fumarate hydratase (FH) in the tricarboxylic acid cycle leads to TCA circulatory dysfunction and mitochondrial metabolic defects in many cancers [12]. This result indicates that the tricarboxylic acid cycle may be also involved in the progression of OS.

### 2.3 Pentose phosphate pathway

The pentose phosphate pathway begins with the intermediate product of glycolysis, glucose-6-phosphate. This pathway does not result in ATP; however, two important products, phosphoribose and NADPH, are generated. The former is an essential substance for synthesizing nucleic acids and the latter an important antioxidant substance. Because of the accelerated metabolism, the ROS level of cancer cells is usually higher than that of normal cells. NADPH produced by pentose phosphate pathway can help tumour cells fight against oxidative stress damage caused by ROS and generate high levels of nucleotides for DNA synthesis and repair [13]. This result indicates that the pentose phosphate pathway may be also involved in the progression of OS.

## 3 Target molecules affecting glycometabolism in OS development

### 3.1 Glycolysis

#### 3.1.1 Glucose transporters

Glucose transporters (GLUT) are transmembrane proteins that regulate the entry of extracellular glucose into cells, mainly involved in processes such as glucose metabolism, inflammatory response, and immune response. Multiple studies have shown that GLUT1 exhibits a high expression trend in the development of various tumours (oesophageal cancer, prostate cancer, lung cancer) and can affect tumour progression by mediating glycolysis, tumour cell proliferation, and immune cell infiltration [14], [15], [16]. In addition, in the study of the clinical and pathological significance of overexpression of glucose transporter-1 in human osteosarcoma, immunohistochemistry was performed to detect Glut–1 protein expression in 51 paired osteosarcoma specimens and adjacent non–cancerous tissues, and reverse transcription– quantitative polymerase chain reaction analysis was performed to examine Glut–1 mRNA expression levels in 6 pairs of these tissues. The results showed that Glut–1 expression levels were significantly associated with age, tumor–node–metastasis stage, lymph node metastasis and survival. It is thus clear that GLUT1 also shows a significant trend of high expression in OS patients’ tissues, and its expression level is closely related to the invasion and metastasis of OS, which may be a key target molecule for judging the quality of life of OS patients [17]. The expression of GLUT1 is closely related to non-coding RNA. Yuan et al. [18] found that the expression level of miR-150 was significantly reduced in OS cell lines (G-292, Saos2, MG-63, HOS, and U2OS) than that of the normal bone cell line hFOB19. Subsequently, the author selected OS cell lines (HOS and U2OS) with significant levels of decline for research and found that GLUT1 is a direct target of miR-150, which downregulates the expression of GLUT1. The expression of miR-150 in OS cells is downregulated, and the resulting regulation of GLUT1 which affects the glucose uptake of OS cells [18]. As a result, the glycolysis rate as well as the and extracellular acidification rate of OS cells are accelerated, which in turn causes the progression of OS to accelerate. Geng et al. [19] found that miR-140-5p was downregulated in OS and there was a significant reverse correlation between microRNA-140-5p and GLUT1 expression in the serum of OS patients. When the expression of microRNA-140-5p is downregulated, it promotes the expression of GLUT1, thereby promoting the growth of OS cells [19]. In addition, microRNA-140-5p is also significantly correlated with tumour size [19]. Research has found that miR-328-3P is downregulated in OS cell line (U2OS, Soas, and MG63), especially in MG63 cell line. Therefore, the author selected MG63 cell line for research, this study confirms that miR-328-3P has been found to be downregulated in OS cells, which significantly increases the proliferation ability of OS cells [20]. Further research has shown that GLUT1 is a direct target of miR-328-3P, and these genes expression is negatively correlated [21]. Downregulation of miR-328-3P expression promotes the expression of GLUT1 and accelerates glucose uptake by OS cells [21]. Subsequently, a new human recombinant bioengineered miRNA reagent (hBERA) carrying warhead miR-328-3p was designed by using htRNASER/pre miR-34a as the carrier and replacing the miR-34a double stranded body with the miR-328-3p sequence [21]. hBERA/miR-328 was found to significantly inhibit the proliferation ability of OS cells after the combination of hBERA/miR-328-3p and cisplatin or amycin was used to treat OS [21]. It is suggested that hBERA/ miR-328-3p has a strong synergistic effect with chemotherapy drugs in controlling the occurrence and development of OS. Chen et al. [22] found that the expression levels of miR-522-3p and GLUT1 were significantly increased in OS tissue and in OS cell lines (U2OS and MG-63), and the expression levels of the two were significantly correlated. Further research has found that overexpression of miR-522-3p leads to upregulation of GLUT1 expression, thereby promoting glucose uptake and OS cell growth [22]. In addition, lncRNA is also involved in the expression of GLUT1 in OS tissues and cells. The expression level of lncRNA HAND2-AS1 was found to decrease in both OS tissue, patient serum, and in OS cell lines (MG-63 and SAOS-2)[23]. The expression of HAND2-AS1 is significantly correlated with tumour size, and low expression upregulates GLUT1 expression, increases glucose uptake, and promotes OS cells proliferation [23]. Yu et al. [24] found that STC2 is highly expressed in OS tissue and in five OS cell lines (U2OS, U2R, MG63, 143B, ZOS), and STC2 can promote the expression of GLUT1, accelerate glucose uptake rate, and stimulate lactate production, thereby promoting the proliferation and metastasis of OS cells. Li et al. [25] found that Ctsk is expressed in chondrogenic cells and periosteal stem cells, and the loss of Trp53/Rb1 in Ctsk expressing cells was significant, increasing the expression and activity of Yes related protein (YAP). The YAP/TEAD1 complex binds to the glucose transporter 1 (GLUT1) promoter, ultimately upregulating GLUT1 expression, the upregulation of GLUT1 expression leads to overactive glucose metabolism and promotes the occurrence and development of OS [25]. The authors [26] also found that P53 mutations in SaOS-2 OS cell line impair the inhibitory effect of wild-type p53 on the transcriptional activity of GLUT1 and GLUT4 gene promoters, thereby increasing the expression level of GLUT protein and the ability of tumour cells to uptake glucose, leading to faster proliferation of tumour cells. It can be seen that GLUT1 is closely related to the occurrence and development of OS, and non-coding RNA (miR-150, microRNA-140-5p, miR-328-3P, miR-522-3p, lncRNA HAND2-AS1) and some small molecules (STC2, Ctsk, p53) change the rate of glucose metabolism by regulating the expression of GLUT1, thereby affecting the occurrence and development of OS. In addition, GLUT1 inhibitors have a strong synergistic effect when combined with chemotherapy drugs [21]. Therefore, GLUT1 may be a key target molecule that affects the occurrence and development of OS, and its mechanism of action needs more research to confirm. Further exploration of the role of GLUT1 in the occurrence and development of OS, as well as additional work on related GLUT1 inhibitors, is expected to lead to effective strategies for treating OS.

#### 3.1.2 Hexokinase

Hexokinase (HK) is an important key enzyme involved in glucose metabolism, which mainly catalyzes the phosphorylation of glucose to produce glucose 6-phosphate (G-6-P). In mammals, there are four main isomers of hexokinase: HK1, HK2, HK3 and HK4 [27]. HK2 is located at the core of the growth and survival process of tumour cells. HK2 can promote glucose metabolism to meet the energy needs of tumour cells, inhibit the accumulation of ROS and Ca^2+^ in mitochondria, and increase the adaptability and survival of tumour cells [27]. Research has shown that HK2 is upregulated in various tumours and is involved in the occurrence and development of tumours (cervical cancer, liver cancer, prostate cancer) [28], [29], [30]. Sun et al. [31] found that miR-615 was downregulated in OS tissue, and HK2 was a direct target of miR-615, with a negative correlation between the expression of the two. In OS, the downregulation of miR-615 promotes the expression of HK2, thereby promoting the proliferation and metastasis of OS cells [31]. Liu et al. [32] predicted the potential downstream target of miR-185 and found that the rate limiting enzyme HK2 of glycolysis may be the downstream target of miR-185. Through luciferase labelling analysis, it was found that miR-185 was bound to the 3′ untranslated region of HK2 mRNA, and the expression of miR-185 was significantly downregulated in OS tissues and OS cell lines (HOS, U2OS, Saos-2, MG-63), especially in U2OS and Saos-2 cell lines. Therefore, the author selected U2OS and Saos-2 cell lines for research, this study confirms that there was

a reverse correlation between the expression levels of miR-185 and HK2 was reported [32]. In OS, the downregulation of miR-185 promotes the expression of HK2 and the rate of glycolysis, accelerating the progression of OS [32]. In addition, it was also found that miR-185 had inhibitory effects on glucose consumption and lactate production in OS cells, whereas overexpression of HK2 in OS cells significantly weakened the inhibitory effect of miR-185 on OS [32]. Therefore, miR-185 may become an effective target for the treatment of OS. Song et al. [33] found that lncRNA PVT1 is overexpressed in OS, and PVT1 promotes the expression of HK2 in OS cells by acting as a competitive endogenous RNA (ceRNA) for miR-497, leading to a significant increase in glucose consumption and lactate production in OS cells. Liu et al. [34] reported that the expression of lncRNA FEZF1-AS1 and CXCR4 was significantly upregulated in OS tissue and OS cell lines (Saos-2 and HOS). In contrast, the expression of miR-144 was significantly downregulated [34]. Further research [34] has found that lncRNA FEZF1-AS1 can act as the ceRNA of miR-144, significantly upregulating the expression of CXCR4 and subsequently significantly upregulating the expression of HK2, which ultimately promotes the occurrence and development of OS. Long non-coding RNA (lncRNA) taurine up-regulated gene 1 (TUG1) is considered to be an oncogene in OS [35]. Han et al. [35] found that, compared to normal bone cell lines, lncRNA TUG1 was overexpressed in OS cell lines (Saos-2, U2OS, HOS和MG63), especially in U2OS and Saos-2 cell lines. Therefore, the author selected U2OS and Saos-2 cell lines for research, the results confirm that lncRNA TUG1 overexpression promoted the survival ability of OS. Further research [35] has found that overexpression of TUG1 leads to an increase in HK2 protein levels, promotes glucose consumption, and accelerates lactate production. When HK2 decreases, it also weakens the effect of TUG1 overexpression on glycolysis in OS cells [35]. Therefore, the effect of lncRNA TUG1 on the survival ability of OS cells may be related to glycolysis, and HK2 may be a key enzyme that affects the rate of glycolysis reaction by TUG1. LncRNA-SARCC is down-regulated in OS cell lines (HOS, Saos-2, MG63, 143B, U2OS). For the establishment of the cisplatin-resistant OS cell line(SaoS-2), and negative correlated with cisplatin resistance [36]. Further studies showed that lncRNA SARCC could up-regulate the expression of miR-143. HK2 is the direct target gene of miR-143 [36]. In OS cells, overexpression of miR-143 significantly decreased the expression of HK2 protein [36].Therefore, lncRNA SARCC negatively regulates the expression of HK2 protein through miR-143 in OS cell lines. In OS cells, this negative regulatory effect was weakened, which indirectly led to the enhancement of glycolysis and the increase of cisplatin resistance in OS cells [36]. CircFAT (e2) has been shown to be upregulated in OS cell lines (SW1353, U2OS, MG-63), the expression of CircFAT1E2 in SW1353 and U2OS cell lines is more than five times that of normal human cells, and circFAT (e2) enhances the expression of HK2 by acting as a competitive endogenous RNA (ceRNA) for miR-181b, thereby promoting the proliferation and migration of OS cells [37]. When knocking down circFAT (e2), the proliferation ability of OS cells significantly decreases [37]. Thus, the circFAT1 (e2)/miR-181b/HK2 axis may be a potential target for the treatment of OS. Yang et al. [38] found that circ-CTNNB1 is highly expressed in the nucleus of OS and interacts with RBM15, after which it is modified with N6-methyladenosine (m6A) to promote the expression of HK2, thereby promoting the glycolysis process and accelerating OS progression. Some investigators [39] have also found that, in OS tissue and OS cell lines, (143B, HOS, MG-63, SJSA-1, SaoS-2, and U2OS), Circle_0,016,347 expression was upregulated, with 143B and MG-63 cell lines showing significant effects. Therefore, the author selected 43B and MG-63 cells for subsequent research and found that circle_0016347 and KCNH1 were upregulated, and miR-1225-3p was downregulated in OS cell lines (43B and MG-63). Circ_0016347 regulates KCNH1 expression by acting as a competitive endogenous RNA (ceRNA) for miR-1225-3p. Circ_0016347 upregulates HK2 expression through the miR-1225-3p/KCNH1 axis, promoting the progression of OS [39]. Studies [40] found that circ_0000591 is highly expressed in OS cell lines (U2OS, HOS, SaoS2, and MG63) and promotes HK2 expression by acting as a molecular sponge of miR-194 −5p. Overexpression of HK2 depressed the inhibitory effect of miR-194-5p on the glycolysis of OS cells, and promoted the proliferation and migration of OS cell lines (U2OS and MG63)[40]. Additional research [41] has shown that from 50 % to 60 % of locally advanced solid tumours may exhibit hypoxia and/or hypoxic tissue regions. In hypoxic conditions, the expression of HIF-1 α is significantly elevated, with the result that tumour proliferation and migration are accelerated, negatively affecting the prognosis of tumour patients [41]. In hypoxic OS cells, elevated expression of HIF-1 α leads to upregulation of Angptl2, which promotes the expression of HK2, which in turn promotes OS cell proliferation, metastasis, angiogenesis, and glycolysis [42]. It has been reported that that ROCK2 is upregulated in OS tissue and OS cell lines (U2-OS, Saos-2, 143B, MG-63).The results confirm that upregulates HK2 expression by activating the PI3K/AKT signaling pathway, promoting aerobic glycolysis [43]. Considering the results of these studies, we can conclude that HK2 is closely related to the occurrence and development of OS. Furthermore, non-coding RNA (miR-615, miR-185, lncRNA PVT1, lncRNA FEZF1-AS1, lncRNA TUG1, lncRNA-SARCC, circFAT (e2), circ-CTNNB1, circ_0016347, circ_0000591) and some small molecule substances (Angptl2, ROCK2) can alter the glucose metabolism rate by regulating the expression of HK2, thereby affecting the proliferation and metastasis of OS. Further exploration of the mechanism of action of HK2 in the occurrence and development of OS and the discovery of related HK2 inhibitors would help in the development of treatment strategies for OS.

#### 3.1.3 Phosphofructokinase 1

Phosphofructokinase 1 (PFK-1) is another key enzyme involved in the glycolysis pathway, mainly catalysing the conversion of fructose 6-phosphate to fructose 1,6-diphosphate (F-1, 6-BP). PFK-1 activity is regulated by a number of metabolites, such as adenosine triphosphate (ATP), adenosine diphosphate (ADP), fructose 2,6-diphosphate (F-2, 6-BP), and others. Among them, F-2,6-BP is the most effective allosteric activator of PFK-1, which is a product catalysed by 6-phosphate fructose kinase 2/fructose diphosphatase-2 (PFK-2/FBPase-2) (PFKFB-2). PFKFB-2 belongs to the 6-phosphate fructose kinase 2/fructose diphosphatase (PFKFB) family. Research has confirmed that PFKFB3 and PFKFB4, members of the PFKFB family, are overexpressed in many malignant tumours [44]. However, there is currently no relevant research on whether PFKFB-2 is highly expressed in malignant tumour tissues [44]. It remains to be seen whether the PFKFB family could be a key target for regulating glycolysis in malignant tumour tissue and an effective target molecule for the treatment of malignant tumours. It has been confirmed that the downregulation of miR-26b expression in USOS OS cell line weakens the inhibitory effect on PFKFB3, thereby enhancing glycolysis [45]. PFKM is a known subtype of PFK in humans, Jia et al. [46] found that in OS, lncRNA XLOC_005950 is upregulated and acts as a molecular sponge for miR-542-3p, upregulating the expression of downstream target gene PFKM, thereby promoting glycolysis rate and tumour progression. Other studies [47] have found that the high expression of ROCK2 in OS tissue can upregulate the expression of PFKFB3 by regulating its ubiquitination and degradation, thereby inducing extensive proliferation of OS and promoting its metastasis. However, because neither the expression of PFK-1 nor the rate of glycolysis was measured in this experiment, we can only speculate that the role of ROCK2 in OS may be related to glycolysis. Lysine specific histone demethylase 3A (KDM3A) is an effective histone modifier involved in the progression of various malignant tumours [48]. Research [49] further confirms that KDM3A is highly expressed in OS tissues and OS cell lines [MG63 (CL-0157), Saos-2 (CL-0202), U2OS (CL-0236), HOS]. This study confirms that promotes the expression of PFKFB4 by regulating SP1, thereby promoting glycolysis and tumour proliferation. In addition, the upregulated expression of SLIT2 and ROBO1 in OS cell lines（Saos-2 and U-2OS）can promote the expression of PFKFB2 by activating the SRC/ERK/c-MYC pathway, thereby promoting the Warburg effect in OS cells, which ultimately promotes OS cell proliferation and inhibiting apoptosis [50]. The expression of deubiquitinase USP33 was significantly up-regulated in OS cell lines (143B, U2-OS, Saos-2 and MG-63), and the expression of PFKFB3 was stabilized through the ubiquitin–proteasome pathway, which increased ECAR and decreased OCR in OS cells [51]. It is suggested that USP33 promotes aerobic glycolysis in OS cells and inhibits mitochondrial respiration. In addition, studies [52] have also found that ubiquitin like protein FAT10 up-regulates PFKFB3 expression by stabilizing EGFR, however the up-regulated expression of PFKFB3 could promote the glycolysis and growth of OS. COPS3 [53] is a newly discovered oncogene. In OS cell lines, the expression of COPS3 (COP9 signaling complex subunit 3) is significantly higher than that in normal tissues, and COPS3 promotes the mRNA and protein levels of PFKFB3 and increases the glycolysis rate of OS cells [53]. It can be seen that PFKFB is closely related to the progress of OS, but there is currently no relevant report on whether PFKFB participates in the progress of OS by regulating PFK-1. Non-coding RNA (miR-26b, lncRNA XLOC_005950) and some small molecular substances (ROCK2, KDM3A, deubiquitinase USP33, ubiquitin like protein FAT10, COPS3) can indirectly or directly regulate the expression of PFKM or PFKFB, which changes the rate of glucose metabolism, thereby affecting the progress of OS. However, whether the above non-coding RNA and small molecular substances can affect the expression of PFK-1 (thus participating in the glycolysis process of OS) has not been reported. Therefore, the mechanism of PFK-1 in OS is expected to become a hot topic in OS research.

#### 3.1.4 Aldolase (ALDOA)

The main role of aldolase in the glycolysis pathway is to cleave F-1, 6-BP into 3-phosphate glyceraldehyde and phosphate dihydroxyacetone. ALDOA is highly expressed in OS cells, and its high expression promotes the proliferation and migration of OS cells, significantly reducing the rate of tumour cell apoptosis [54]. Further research [55] has found that the expression of IncRNA KCNQ1OT1 is increased in four OS cell lines (U-2OS, 143B, MG63, and Saos-2), which is a competitive endogenous RNA (ceRNA) of miR-34c-5p, which can weaken the latter's targeted inhibition of ALDOA and ultimately promote OS proliferation, especially in the U-2OS and 143B cell lines. There are few reports on ALDOA in OS related studies. ALDOA may be a key target molecule that affects the occurrence and development of OS, and its mechanism of action needs more research to confirm.

#### 3.1.5 Phosphoglycerate kinase 1

The role that phosphoglycerate kinase 1 (PGK1) plays in the glycolysis pathway is as a catalyst in the conversion of 1, 3-diphosphoglycerate to 3-phosphoglycerate and the conversion of ADP to ATP. PGK1 is highly expressed in various tumours (liver cancer, renal clear cell carcinoma, lung adenocarcinoma), and its high expression within tumour cells promotes tumour cell proliferation and metastasis [56], [57], [58]. However, high expression of PGK1 in the extracellular matrix can inhibit tumour angiogenesis through the production of angiostatin, which plays a role in inhibiting tumour proliferation [59]. The expression of lncRNA HCG18 has been found to be significantly increased in OS cell lines(MG63, MNNG-HOS, U-2OS, 143B) [60]. HCG18 promotes PGK1 mediated glycolysis by acting as a competitive endogenous RNA (ceRNA) for miR-365a-3p, thereby promoting the proliferation of OS cells [60]. In addition, Yang et al. [61] found that the expression of circ-CTNNB1 was significantly up-regulated in OS cell lines (143B, HOS, MG-63, SJSA-1, Saos-2 and U2OS). Interaction between circ-CTNNB1 and RBM15 could up-regulate the expression of HK2 and PGK1 through N6-methyladenosine (m6A) modification, which was promoted aerobic glycolysis and activated the progression of OS [61]. Research [62] has found that YTH N6 methyladenosine RNA binding protein 3 (YTHDF3) is significantly expressed in OS cell lines (MG63, Saos-2), and YTHDF3 enhances the stability of PGK1 mRNA through m6A-dependent pathways, promoting aerobic glycolysis and proliferation of OS cells. Evidently, lncRNA HCG18, circ-CTNNB1 and YTHDF3 can alter the glucose metabolism rate of OS cells by regulating the expression of PGK1, playing an important role in the progression of OS. PGK1 may be a key target molecule that affects the occurrence and development of OS and is expected to become an effective target for screening the efficacy of OS drugs.

#### 3.1.6 Pyruvate kinase 2

Pyruvate kinase 2 (PKM2) is another key enzyme in glucose metabolism. Its role in the glycolysis pathway is mainly to catalyse the dehydration of 2-phosphoglycerate to produce phosphoenolpyruvate (PEP). There is increasing evidence that PKM2 plays a crucial role in promoting the progression of cancer (bladder cancer, hepatocellular carcinoma, breast cancer) [63], [64], [65]. Overexpression of PKM2 predicts poor prognosis in patients with OS [66]. Knockout of PKM2 has been shown to inhibit the proliferation and migration of OS cells, while inducing apoptosis of OS cells [67], providing a basis for PKM2 inhibitors to treat OS. Chen et al. [68] found that miR-491-5p expression is downregulated in OS tissues and cell lines (KHOS, LM7, U2OS and MG-63), and downregulation of miR-491-5p expression enhances PKM2 expression, thereby promoting tumour cell proliferation. In contrast, overexpression of miR-491-5p inhibits the expression of PKM2 [68]. Yuan et al. [69] also found that miR-1294, which is downregulated in OS cell lines (MG63 and HOS), can also promote tumour cell proliferation and migration by upregulating PKM2 expression. Pu et al. [70] found that overexpression of lncCCAT1 in OS cell lines (HOS, KHOS-240S, and U2OS) can activate PKM2, which not only enhances the Warburg effect by upregulating the c-myc gene, but also promotes adipogenesis by upregulating SREBP2 phosphorylation, thereby inducing OS. IRF7 belongs to the transcription factor family of interferon regulatory factors (IRFs). In a recent study [71], it was reported that IRF7 is significantly downregulated in OS tissues due to abnormal methylation. Further study [71] has found that low expression of IRF7 increases the protein level of PKM2 and enhances the glycolytic ability of OS cells, specifically manifested in increased glucose uptake, lactate production, increased ECAR, and decreased OCR. It also inhibits apoptosis of OS cells and promotes the occurrence and development of OS [71]. There is evidence that TRIM58 is downregulated in human OS tissue [72]. Further research has found that TRIM58 may inhibit the activity of PKM2 by enhancing its polyubiquitination in OS cells [72]. In OS, TRIM58 is significantly downregulated, weakening its inhibitory effect on PKM2 and promoting the glycolysis rate and proliferation of OS cells [72]. The above results suggest that PKM2 is closely related to OS, and non-coding RNA (miR-491-5p, miR-1294, lncCCAT1) and some small molecules (IRF7, TRIM58) can change the rate of glucose metabolism by regulating the expression of PKM2, and PKM2 plays a key role in the progression of OS.

#### 3.1.7 Lactate dehydrogenase

Lactate dehydrogenase (LDHA) catalyses the hydrogenation of pyruvate to produce lactic acid, and the lactic acid produced by glycolysis contributes to the progress of malignant tumours to a large extent. LDHA is highly expressed in many cancers and is a key participant in the progress of cancer (thyroid papillary cancer, prostate cancer, breast cancer) [73], [74], [75]. LDHA promotes malignant progression of tumours by increasing the production of lactic acid, accelerating glycolysis, regulating the production of reactive oxygen species, and regulating many cancer-related proteins [76]. It has been shown that miR-323a-3p is significantly downregulated in OS tissue and participates in the growth and metastasis of OS [77]. miR-323a-3p binds to 3′-UTR of LDHA and inhibits the expression of LDHA in OS cell lines(U2OS, SAOS2, MG63, HOS, 143B) [77]. LDHA may be a direct target of miR-323a-3p [77]. By downregulating the expression of miR-323a-3p, its inhibitory effect on LDHA is weakened, thereby promoting glycolysis and accelerating OS progression [77]. In other studies [78], [79], it has also been shown that LDHA may also be a direct target of miR-33b and miR-329-3p. Downregulating the expression of miR-33b and miR-329-3p can also weaken their inhibitory effect on LDHA, promote glycolysis and OS progression [78], [79]. Hu et al. [80] found that the expression of circ-CNST was significantly increased in human OS tissue and OS cells, and significantly inhibited the expression of miR-578. Further research [80] found that under the condition of miR-578 deficiency, the expression of LDHA was upregulated, promoting the rate of glycolysis in OS cell lines (HOS, MG63, 143B, U2OS) and reducing the rate of cell apoptosis. The results showed that LDHA was a direct target of miR-578, and its regulatory effect on glycolysis may require the assistance of circ-CNST. Huo et al. [81] found that circle_0056285 expression is significantly upregulated in OS tissue and OS cell lines(143B, MG63, U2OS, HOS), and TRIM44 expression is increased by acting as a competitive endogenous RNA (ceRNA) for miR-1244, thereby accelerating glycolysis by promoting the expression of LDHA and HK2, ultimately inducing OS and accelerating its progression. CircATRNL1, which is significantly expressed in OS tissue and cell lines (U2OS, Saos-2, MG63), also promotes LDHA expression by acting as a competitive endogenous RNA (ceRNA) for miR-409-3p, further promoting glycolysis and the progression of OS [82]. Jiang et al. [83] found that KDM6B expression in OS patients was significantly upregulated. KDM6B directly mediated the H3K27me3 demethylation of LDHA, with the result that the expression of lactate dehydrogenase LDHA was increased in OS cells, thereby promoting tumour metastasis [83]. It can be seen that LDHA is involved in the progression and metastasis of OS, and this effect is to regulate the expression of LDHA through non-coding RNA (miR-323a-3p, miR-33b, miR-329-3p, circ-CNST, circ_0056285, circATRNL1) and small molecule KDM6B. In this way, the glucose metabolism rate of OS is altered.

### 3.2 TCA

#### 3.2.1 Pyruvate dehydrogenase complex

The function of the pyruvate dehydrogenase complex (PDC) is to catalyse the production of acetyl CoA from pyruvate, which enters the tricarboxylic acid cycle. In tumours, in most cases, pyruvate is converted into lactate in the cytoplasm [84]. In rare cases, pyruvate enters the mitochondria and is metabolized there, with the result that PDC is downregulated in OS [84]. Pyruvate dehydrogenase kinase (PDK) is a negative regulator of PDC and inhibits PDC activity [84]. The pyruvate dehydrogenase kinase (PDK) family, including PDK1, PDK2, PDK3, and PDK4, is closely related to tumour progression [84]. When PDC activity is inhibited, lactate accumulation and angiogenesis are promoted, which is beneficial for tumour cell proliferation [84]. There is evidence [85] that PDK inhibitors can not only block aerobic glycolysis but also promote caspase mediated cell apoptosis and have an inhibitory effect on tumours, which indicates that PDK inhibitors have great potential in the treatment of OS. It has been reported [86] that significantly downregulated miR-379 in OS tissues and cell lines (MG-63, U2OS, SOSP-9607, SAOS-2) can significantly upregulate PDK1 expression, and high expression of PDK1 promotes tumour cell proliferation and migration, indicating that PDK1 is a direct target of miR-379. Additional research [87] has found that the low expression of miR-15b-5p in OS promotes the expression of PDK4, which in turn affects the Warburg effect in OS cell lines (MNNG-HOS, Saos-2 and MG63 cells) and the proliferation of OS cells. Both miR-379 and miR-15b-5p can affect the progression of OS by regulating the expression of PDK. However, in neither of the above experiments was the expression level of PDC measured. Therefore, we can only speculate that the role of miR-379 and miR-15b-5p in OS is related to PDC. Although there is evidence that PDK inhibitors have great potential for treating OS, there is still a lack of data pertinent to the underlying mechanism.

#### 3.2.2 Isocitrate dehydrogenase

The role of isocitric acid dehydrogenase (IDH) is to catalyse the oxidative decarboxylation of isocitric acid to α-ketoglutarate. Of the three subtypes of IDH, IDH1 mainly exists in peroxisome and cytoplasm, and IDH2 and IDH3 are located in the mitochondria [88]. IDH1 and IDH2 enzymes catalyse oxidative decarboxylation of isocitric acid α-ketoglutaric acid [88]. IIDH1 and IDH2 are common mutated metabolic enzymes in cancer, and multiple studies have shown that they mutate in cancer, and mutant IDH can accelerate tumour progression, which indicates that mutant IDH is carcinogenic [89], [90], [91]. The expression of wild-type IDH1 is downregulated in OS tissue, and its downregulation can enhance the Warburg effect [92]. When wild-type IDH1 is upregulated, it has anti proliferation and proapoptotic effects on OS cells and inhibits tumour metastasis [93]. Therefore, upregulating the expression of wild-type IDH1 may be a potential new therapy for OS. It has been shown that HIF-1 α is highly expressed in OS tissue and cell lines (MG-63, 143B) [93]. HIF-1 α can increase the incidence rate of OS by reducing the wild-type IDH1 expression [93]. It can be seen that in-depth study of the molecular mechanism of IDH in the progression of OS is a good strategy for tumour drug screening.

#### 3.2.3 Succinate dehydrogenase

Succinate dehydrogenase (SDH) catalyses the dehydrogenation of succinate to fumarate. The SDH molecule binds to the inner mitochondrial membrane. As such, SDH is considered to be a classic mitochondrial enzyme involved in citric acid cycle and electron transfer. In cancer, SDH reduction is mainly achieved through subunit mutation or its promoter methylation [94], [95]. The reduction of SDH leads to the accumulation of succinic acid, which in turn promotes tumour metastasis and angiogenesis, which is crucial in the progression of cancer [96]. The expression of transforming growth factor-β (TGF-β) is stronger in chemotherapy-resistant OS patients [97]. Furthermore, upregulation of TGF-β can downregulate the expression of SDH by inhibiting the expression of transcription factor STAT1 in chemotherapy induced OS patients, leading to the accumulation of succinic acid, thereby inhibiting its tricarboxylic acid cycle and promoting HIF-1 α [97]. The overexpression of SDH ultimately exacerbates the chemotherapy resistance of OS patients, leading to the progression or deterioration of the disease [97]. These results suggest that SDH plays an important role in the induction of chemoresistance in osteosarcoma, which is closely related to the decrease of tricarboxylic acid cycle in glucose metabolism.

### 3.3 Pentose phosphate pathway

#### 3.3.1 Glucose 6-phosphate dehydrogenase

Glucose 6-phosphate dehydrogenase (G-6-PD), a key enzyme involved in the pentose phosphate pathway, is crucial for the production of NADPH. Recent studies have shown that G-6-PD is upregulated in various cancers and is associated with tumour progression and tumour chemotherapy resistance [98], [99], [100]. The main function of G-6-PD is to provide sufficient NADPH to maintain redox homeostasis [101]. In addition, G-6-PD can also serve as an angiogenic factor to promote angiogenesis, provide nutrition for tumour cells, and promote tumour cell growth [101]. It has been reported that the expression of IncRNAOR3A4 is abnormally elevated in OS tissues and cell lines (MG-63, SaoS-2, SJSA-1, G-292), which downregulates the expression level of miR-1207-5p and weakens the inhibitory effect of the latter on G-6-PD mRNA [102]. Therefore, IncRNAOR3A4 can be assumed to promote the expression of G-6-PD and the production of NADPH, inhibiting cell apoptosis caused by oxidative stress. G-6-PD plays a very important role in the progression of OS. However, there is limited research on the specific mechanisms. Further exploration of the mechanism of G-6-PD in the occurrence and development of OS may lead the way to more effective treatment of OS.

**Fig. 1:**
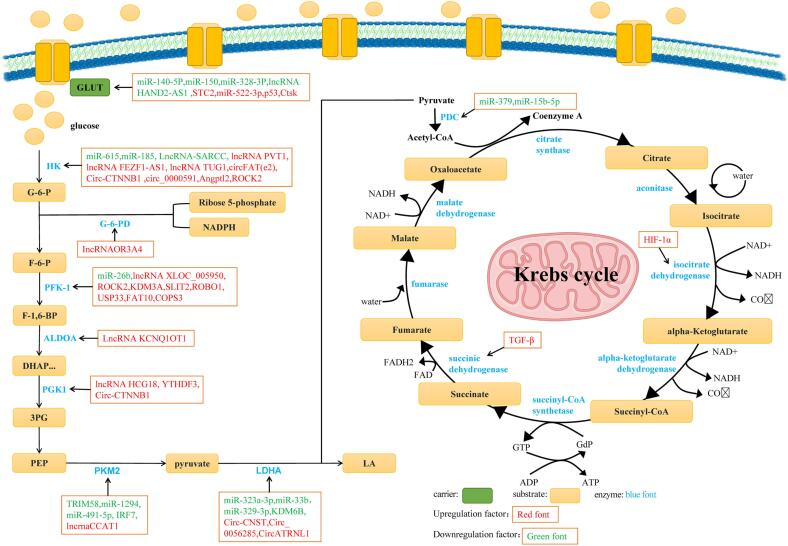
Non-coding rna and small molecule substances regulate glucose uptake and glycolysis, tricarboxylic acid cycle and pentose phosphate pathway. HK, PFK-1, ALDOA, PGK1, PKM2, LDHA, etc. in glycolysis pathway can participate in OS progression under the regulation of some non-coding RNA and small molecule substances. G-6-PD in the pentose phosphate pathway is involved in the progression of OS under the influence of lncRNAOR3A4. In the tricarboxylic acid cycle pathway, PDC is involved in the progression of OS under the influence of miR-379, miR-15b-5p, and IDH is adjusted by HIF-1 α, SDH is adjusted by TGF-β, these also participate in the progression of OS. GLUT1: glucose transporter 1; HK: hexokinase; G-6-P: Glucose-6-phosphate; F-6-P: Fructose-6-phosphate; PFK-1: Phosphate fructose kinase 1; F-1, 6-BP: fructose-1, 6-diphosphate; ALDLA: aldolase; DHAP: dihydroxyacetone phosphate; PGK1: phosphoglycerate kinase 1; 3PG: triphosphoglycerate; PEP: phosphoenolpyruvic acid; PKM2: pyruvate kinase 2; LDHA: lactate dehydrogenase; LA: Lactic acid; PDC: Pyruvate dehydrogenase complex. The glycolysis pathway and pentose phosphate pathway occur in the cytoplasm, while the tricarboxylic acid cycle occurs in mitochondria.

In summary, the progression, metastasis, and angiogenesis of OS are closely related to the activation of multiple key enzymes in glucose metabolism pathways such as the glycolysis pathway, pentose phosphate pathway, and tricarboxylic acid cycle, and the regulation of these enzyme activities is regulated by some noncoding small RNAs or other small molecule substances, forming a complex network regulation (Fig. 1). However, to date, the specific mechanisms through which these noncoding small RNAs or other small molecule substances exert this regulatory effect have yet to be clarified. This may be a hot topic in future research on tumour mechanisms and a key target for screening the efficacy of future cancer drugs.

## 4 Signal pathways affecting the process of glucose metabolism in OS cells

The pathogenesis of OS is extremely complex, and multiple signaling pathways may be involved in the regulation of glucose metabolism pathways, thereby regulating the occurrence, invasion, and migration of OS. Understanding the signaling pathways involved in glucose metabolism pathways and their molecular regulatory mechanisms can help reveal the pathogenesis of OS and thus aid future drug development.

### 4.1 mTOR signaling pathway

Mammalian rapamycin target protein (mTOR) is a serine/threonine protein kinase belonging to the phosphoglycoside-3-kinase (PI3K) related kinase family. The activation of the mTOR signaling pathway contributes to the progression of different types of cancer, including OS [103]. After phosphorylation of mTOR, the activated mTOR accelerates OS progression by overexpression of downstream effector factors, namely S6K1, 4EBP1, and eIF4E [103]. Multiple studies [104], [105] have shown that targeting mTOR complexes is an important method for cancer treatment research. It has also been shown that phospholipase and angiotensin homolog (PTEN) are tumour suppressor genes that can negatively regulate the phosphatidylinositol 3-kinase (PI3K)/protein kinase B (AKT)/mTOR pathway [106]. Therefore, exploring ways to enhance the anti-tumour activity of PTEN may lead to a new targeted therapy for OS. Chamomine can inhibit the PI3K/AKT/mTOR signaling pathway, weaken the expression of MMP-2/9, and inhibit the invasion and metastasis of OS [107]. It has been reported that 200 μm arbutin significantly inhibited the activity of OS cells, which resulted in a significant increase in the expression of miR-338-3p and a corresponding significant decrease in the expression of MTHFD1L [108]. After treatment with arbutin, the mTOR pathway activity in OS was inhibited, thereby inhibiting the occurrence and development of OS [108]. Further, it has been demonstrated that lncRNA MALAT1 (metastasis associated lung adenocarcinoma transcript 1) binding to miR-485-3p can significantly downregulate the expression of miR-485-3p [109]. miR-485-3p can also directly target mesenchymal transition factors c-MET and AKT3 and reduce the expression levels of glycolytic related proteins (GLUT1, HK2, and PKM2) and migration related proteins (MMP2 and MMP9) by inhibiting the mTOR pathway [109]. Keratin 17 (KRT17) [110] is a key member of keratin and is dysregulated in various types of cancer. KRT17 is upregulated in OS [110]. KRT17 knockout reduces phosphorylation (p) -AKT, p-mTOR, HIF-1 α and inhibits the Warburg effect. However, the effects of KRT17 knockout on cell proliferation and glycolysis are reversed when p-Akt, p-mTOR or HIF-1 α expression is restored [110]. Therefore, KRT17 is upregulated in OS and can accelerate the progression of OS by activating AKT/mTOR/HIF-1α pathway [110]. PDGFRβ is a membrane receptor strongly expressed in OS cells and vascular parietal cell [111]. Research [111] has found that it is possible to promote aerobic glycolysis of OS cells by activating the PI3K/AKT/mTOR/c Myc pathway. S100 calcium binding protein A10 (S100A10) is another protein molecule overexpressed in OS tissue, which can accelerate the glycolysis process of OS by activating AKT/mTOR signaling, thereby promoting the malignant transformation of OS cells [112]. This suggests that the PI3K/Akt/mTOR signaling pathway may be involved in the glycolysis process of OS, thereby accelerating the proliferation, migration, and invasion of OS cells.

### 4.2 Hippo pathway

The Hippo signaling pathway is an evolutionarily conserved signaling cascade that regulates many biological processes [113]. The mammalian Hippo pathway mainly includes MST1/2, LATS1/2, downstream effectors, transcription co activators YAP, and TAZ [113]. The imbalance of the Hippo signaling pathway can lead to abnormal cell growth and promote tumour development, and some small molecule inhibitors targeting the Hippo signaling pathway have the potential to treat OS [114]. In addition, the Hippo/YAP signaling pathway also plays a role in chemotherapy of OS, with YAP being a potential target for reducing tumour resistance to chemotherapy [115]. It has also been shown that verteporfin (VP) is a specific inhibitor of Yes related protein 1 (YAP1), which inhibits the Hippo pathway by inhibiting the expression of YAP1, thereby achieving the goal of treating OS [116]. In addition, the expression of GLUT3 is significantly upregulated in cells expressing the YAP-5SA mutant, indicating that the activation of the hippo pathway can promote cell glycolysis [117]. In addition, sphingosine 1-phosphate receptor 3 (S1PR3), a member of the GPCR family, and its specific ligand sphingosine 1-phosphate (S1P) were shown to significantly increase expression in OS and promote aerobic glycolysis and OS development through the YAP/c-MYC/PGAM1 axis [118]. YAP is a key molecule in the Hippo pathway, indicating a close relationship between OS glucose metabolism and the Hippo pathway. Evidently, the Hippo pathway is closely related to the glucose metabolism pathway, but the specific regulatory mechanism is not yet clear.

### 4.3 Wnt pathway

The main components of the Wnt signaling pathway are the Wnt protein family, which activates cell membrane receptors through paracrine and autocrine pathways [119]. The structure of the Wnt pathway is based on two signaling pathways: β-chain protein dependent classical pathway (Wnt/ β-catenin pathway) and β-non classical pathways independent of catenin (Wnt/PCP pathway and Wnt/Ca^2+^ pathway) [119]. Nitazoxanide [120] and microRNA-182 [121] can inhibit the growth of OS by inhibiting the Wnt signaling pathway. However, frequent deletions of Wnt signaling pathway genes have been reported in OS [122], indicating that the Wnt signaling pathway may not be active. Accordingly, further studies (both *in vitro* and *in vivo*) are needed on the regulatory role of the Wnt signaling pathway in OS. It was also reported that miR-21-5p expression is upregulated in OS [123]. Knocking out miR-21-5pk can inhibit Wnt/ β-deactivation of catenin signaling, which weakens the Warburg effect and stem cell maintenance in OS cells [123]. It has also been shown that circPVT1 expression is upregulated in OS, and circPVT1 activates the Wnt5a/Ror2 signaling pathway through sponge adsorption of miR-423-5p, promoting glycolysis and metastasis of OS [124]. Circ_0105346 is significantly upregulated in OS cells, acting as a molecular sponge of miR-1182 to up-regulate the mRNA and protein levels of WNT7B, and promote the proliferation, migration and glycolysis levels of OS cells [125]. It can be found that Wnt signaling pathway is closely related to glucose metabolism reprogramming of OS cells, but the relationship between them needs to be further explored and may become a research hotspot.

### 4.4 NF-κB signaling pathway

The NF-κB pathway, including the classical and non-classical NF-κB pathways, function under different activation mechanisms. The classical NF-κB pathway participates in inflammatory response, immune response, cell proliferation, differentiation, and cell survival [126]. The non-classical NF-κB pathway not only regulates the proliferation and migration of B cells but also participates in the development of lymphoid organs and thymus T cells, the generation and maintenance of peripheral effectors and memory T cell, as well as the antiviral immune response and the production of type I interferon [126]. The NF-κB signaling pathway is activated in various human cancers and is closely related to cancer. Research [127] has found that the NF-κB signaling pathway is activated in OS cells and promotes tumour growth and metastasis. NF-κB signaling pathway inhibitors may have anti OS effects [127]. The nuclear factor κB consists of five subunits, namely RelA (called p65), c-Rel, RelB, p50, and p52 [128]. It was found that HK2 is a direct target of NF-κB, especially binding to the p65 subunit of NF-κB. After p65 knockout in OS cells, HK2 expression is down-regulated and Warbug effect is inhibited in OS cells [129]. Therefore, the activated classical NF-κB signaling pathway promotes OS cell glycolysis by upregulating HK2 expression. It can be seen that NF-κB pathway is closely related to OS, but its relationship with glucose metabolism in OS cells remains to be explored.

### 4.5 MAPK signaling pathway

The mitogen activated protein kinase (MAPK) signaling pathway occurs downstream of RAS. This pathway is composed of three kinases: RAF, MEK, and ERK [130]. The MAPK pathway plays different roles in cancer progression. In liver cancer, activation of the MAPK pathway promotes the proliferation and metastasis of liver cancer cells [130]. In gastric cancer, interferon-α (IFN-α), after binding to tumour necrosis factor related apoptosis inducing ligand (TRAIL), upregulates the MAPK pathway and induces apoptosis in gastric cancer cells [131]. It has been reported that the expression of RAF1, MEK1/2, and p-MEK proteins is upregulated in OS cells, indicating the activation of the MAPK pathway in OS [132]. U0126 (MEK1/2 inhibitor) inhibits MAPK signaling and proliferation of OS cells after treating OS cells [132]. Therefore, inhibiting the MAPK signaling pathway is expected to become an effective strategy for OS treatment. Circle_0001721 is upregulated in OS and activates the MAPK7 pathway by acting as a competitive endogenous RNA (ceRNA) for miR-372-3p, promoting glycolysis and the progression of OS [133]. It can be seen that the MAPK pathway is closely related to OS. However, the relationship between it and glucose metabolism in OS cells needs to be explored.

**Fig. 2:**
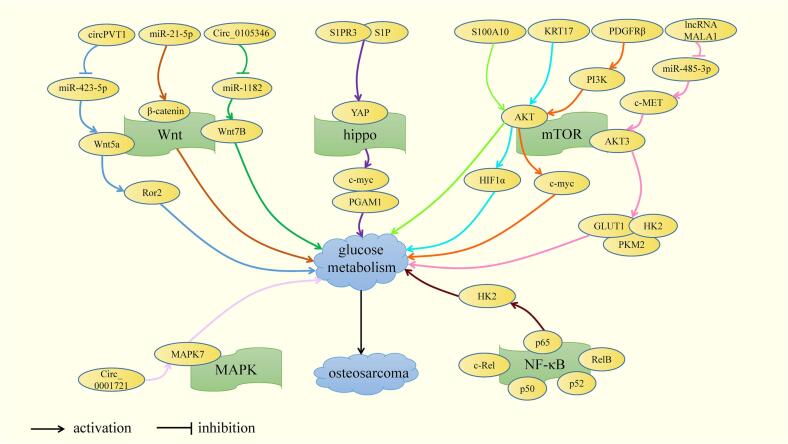
Regulation mechanism of signal pathways on the glucose metabolism in the development of OS. There are 5 signaling pathways, mTOR, Wnt, hippo, NF-κB, MAPK, are related to glucose metabolism in OS cells.

In conclusion,mTOR pathway, hippo pathway, Wnt pathway, NF-κB pathway and MAPK pathway are all involved in the glucose metabolism of OS, among which lncRNA MALAT1, KRT17, PDGFRβ and S100A10 is probably involved in the glycolysis pathway of OS through the activation of mTOR pathway. S1PR3 may promote the glycolysis pathway of OS through the YAP/c-MYC/PGAM1 axisby, which is activated the hippo pathway. miR-21-5p, circPVT1, Circ_0105346 may be involved in the glycolysis pathway of OS through activation of Wnt pathway. The activated classical NF-κB signaling pathway promotes OS cell glycolysis by upregulating HK2 expression. Circ_0,001,721 may participate in the glucose metabolism of OS by activating MAPK pathway. In view of the complexity and network of the pathways, it has not yet been studied whether there are common targets among these pathways to regulate the glycolysis of OS(Fig. 2). A better understanding of the relationship between OS glucose metabolism processes and signaling pathways will help optimize the treatment strategy of OS.

## 5 Potential therapeutic effects of drugs targeting glucose metabolism pathways on osteosarcoma

### 5.1 natural extracts

Studies have shown that regulating glucose metabolism pathways is an effective strategy for treating OS. It was reported that apigenin downregulates the protein expression of vimentin and upregulates the protein expression of E-cadherin in a concentration dependent manner, while also reducing the levels of glucose and lactic acid in OS cells and increase the levels of citrate and ATP [134]. In addition, the expression of PI3K, Akt, and mTOR and the expression of pPI3K and pAkt in OS cells treated with apigenin were significantly down regulated (1 3 4). It has been proved that PI3K/Akt/mTOR is related to glycolysis [135]. Therefore, it is speculated that apigenin inhibits the Warburg effect and cell proliferation of OS cells by inhibiting the PI3K/Akt/mTOR signaling pathway. Research [136] has found that caudatin inhibits cellular glycolysis by reducing the expression of HK2 and LDHA, possibly by downregulating the expression of β-Catenin, Cyclin D1, and C-myc to inhibit the Wnt/β-catenin signaling pathway, thereby affecting the glycolysis process, and ultimately achieving the goal of inhibiting the proliferation and metastasis of OS cells [136]. Resveratrol inhibited the proliferation and glycolysis of OS cells by inhibiting the activity and downstream gene expression of Wnt/β-catenin signaling pathway and enhancing the expression of Cx43 and e-cadherin genes [137].

### 5.2 Chemical compounds

Research [138] has found that specific siRNA knockdown of PKM2 restores the sensitivity of OS stem cells to cisplatin therapy. Metformin reverses the resistance of OS stem cells to cisplatin by inhibiting PKM2 expression and promotes cisplatin induced apoptosis of OS cells [138]. The subunit on the ring of 2-((3-cyanopyridine 2-yl) thio) acetamide A plays a decisive role in the inhibition of human lactate dehydrogenase, making it a potential inhibitor of human lactate dehydrogenase A to inhibit lactate production and activity of OS cells, thus promoting apoptosis of OS cells [139]. The Ag particles released by F-AgÅPs may be the main mediator of F-AgÅPs function, which can significantly inhibit the ability of OS cells to form colonies (this parameter is related to the degree of cancer malignancy), and have a much higher inhibitory effect on tumour cell proliferation than cisplatin [140]. F-AgÅPs induce mitochondrial dysfunction in OS cells, leading to a significant decrease in pyruvate and lactate levels in OS cell culture medium [140]. Further experiments found that F-AgÅPs selectively transfer glucose metabolism from glycolysis to mitochondrial oxidation in OS cells by inhibiting pyruvate dehydrogenase kinase (PDK), inducing reactive oxygen species (ROS) dependent apoptosis and cytotoxicity in OS cells [140]. It was confirmed that the N-formylmorpholine substituent (CDDO-NFM) of the triterpenoid compound CDDO can reduce glucose uptake levels, lactate production, and adenosine triphosphate production in OS cells, thus preventing glycolysis [141]. *In vitro* and *in vivo* experimental results indicate that CDDO-NFM inhibits aerobic glycolysis in OS cells by reducing c-MYC [141]. In addition, CDDO-NFM can also reduce tumour volume and body weight and downregulate the expression of glycolytic enzymes in nude mice [141]. CDDO-NFM inhibits the growth of OS cells *in vitro* and *in vivo*, which may be a promising anti-tumour compound. Studies [142] have shown that pramlintide can enhance the apoptosis and inhibit the proliferation of OS cells by inhibiting the glycolysis of OS cells, when the glycolysis activity is moderately or extremely enhanced in OS cells.Research [143] found that the proliferation of OS cells depends on glutamate. After treatment alone or in combination with metformin, the glutaminase 1 inhibitor (CB-839) inhibits the conversion of glutamine to glutamic acid, leading to glutamine accumulation, inhibition of glycolysis and TCA cycle in OS cells, accompanied by increased fatty acid oxidation and pyrimidine catabolism, thereby inhibiting OS proliferation and metastasis [143].

**Fig. 3:**
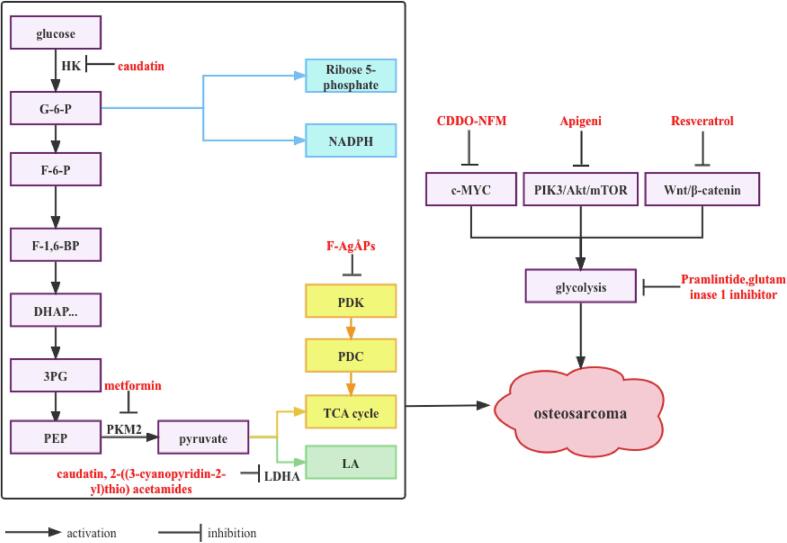
Natural extracts/chemically synthesized substances target the regulation of glucose metabolism pathways in the treatment of OS. caudatin, metformin, CDDO-NFM, pramlintide, resveratrol, glutaminase 1 inhibitor, and apigenin inhibit glycolysis in OS cells; F-AgÅPs promote the tricarboxylic acid cycle in OS cells.

It can be seen that natural extracts (apigenin, caudatin, resveratrol) and chemical compounds (2-(3-cyanopyridyl-2-yl) thio)acetamide, F-AgÅPs, CDDO-NFM, metformin, pramlintide, glutaminase 1 inhibitor) can affect OS progress by targeting the regulation of glucose metabolism (Fig. 3).

## 6 Summary and outlook

Overall, miR-150, microRNA-140-5p, miR-328-3P, miR-522-3p, lncRNA HAND2-AS1, STC2, Ctsk, and p53 regulate the expression of GLUT1; miR-615, miR-185, lncRNA PVT1, lncRNA FEZF1-AS1, lncRNA TUG1, CircFAT(e2), Circ-CTNNB1, Circ_0016347, Angptl2, ROCK2 regulate the expression of HK2;miR-26b, lncRNA XLOC_00595, ROCK2, KDM3A and others can indirectly or directly regulate the expression of PFK; IncRNA KCNQ1OT1 regulates the expression of ALDOA; lncRNA HCG18 and YTHDF3 regulate the expression of PGK; miR-491-5p, miR-1294, LncCCAT1, IRF7, TRIM58 and others can regulate the expression of PKM2; miR-323a-3p, miR-33b, miR-329-3p, Circ-CNST, Circ_0056285, CircATRNL1, and KDM6B regulate the expression of LDHA;IncRNAOR3A4 regulates the expression of G-6-PD;MiR-379 and miR-15b-5p can regulate the expression of PDK; HIF-1 α can adjust the expression of wild-type IDH; TGF-β can regulate the expression of SDH. It can be seen that research on glucose metabolism in OS cells mainly focuses on glycolysis, while research on tricarboxylic acid cycle and pentose phosphate is relatively limited.

**Fig. 4:**
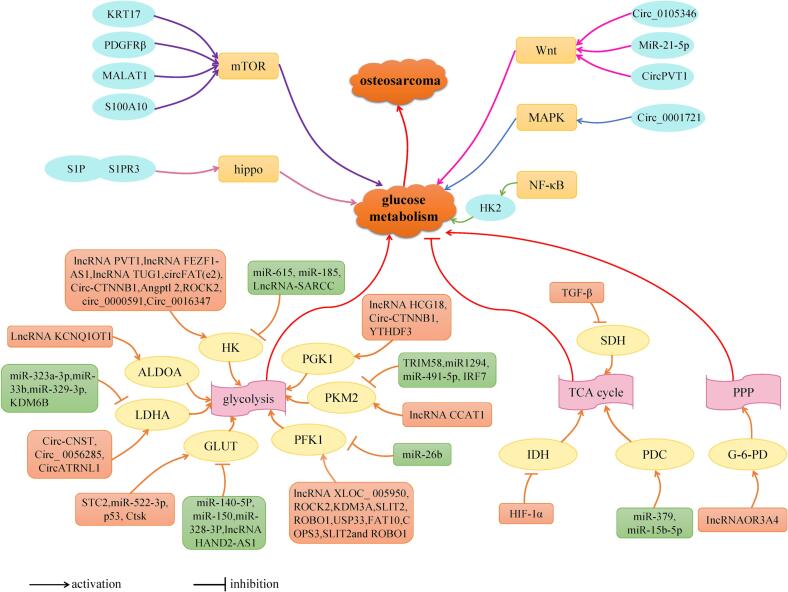
Regulatory mechanism of OS progression and targeted regulatory mechanism of natural extracts and chemical compounds. 11 key molecules, including GLUT, HK, PFK-1, PGK1, PKM2, ALDOA, LDHA, G-6-PD, PDC, IDH, SDH, participate in OS progression under the regulation of non-coding RNA and small molecular substances. It has been shown that five pathways, including mTOR, Wnt, Hippo, NF-κB and MAPK, are related to the glucose metabolism of OS cells. Among them, natural extracts (apigenin, caudatin, resveratrol) and chemical compounds (2 -(3-cyanopyridine −2-yl) thio) acetamide, F-AgÅPs, CDDO-NFM, metformin, pramlintide, glutaminase 1 inhibitor can affect the progression of OS by targeting glucose metabolism. These molecules and pathways can become potential therapeutic targets for OS.

**Fig. 5:**
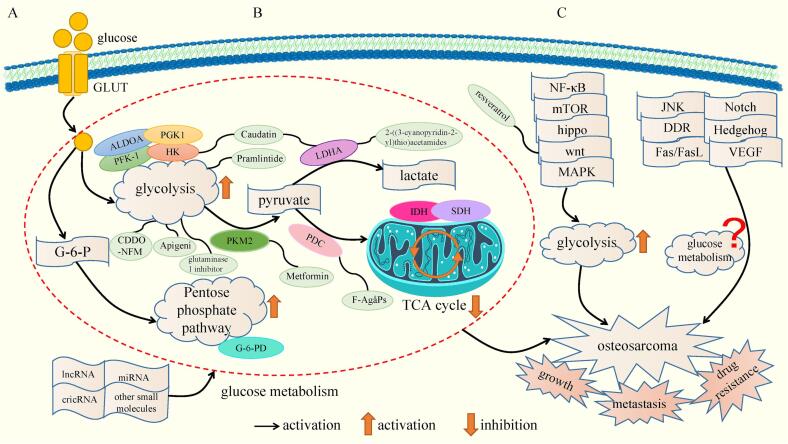
Regulation of glucose metabolism by non-coding RNA, related signaling pathways and natural extracts and chemical syntheses in OS. (A) the regulatory mechanisms of non-coding RNA and small molecules on the activities of key enzymes of glucose metabolism in OS; (B) the regulatory mechanisms of natural extracts and chemical compounds on key enzymes of glucose metabolism in OS; (C) the regulatory mechanisms of related signaling pathways on glucose metabolism in OS.

mTOR pathway, hippo pathway, Wnt pathway, NF-κB signaling pathway and MAPK pathway have been shown to be related to glucose metabolism in OS cells. Studies have shown that lncRNA MALAT1, KRT17, PDGFRβ and S100A10 is probably involved in the glycolysis pathway of OS through the activation of mTOR pathway. S1PR3 may promote the glycolysis pathway of OS through the YAP/c-MYC/PGAM1 axisby, which is activated the hippo pathway. miR-21-5p, circPVT1, Circ_0105346 may be involved in the glycolysis pathway of OS through activation of Wnt pathway. The activated classical NF-κB signaling pathway promotes OS cell glycolysis by upregulating HK2 expression. Circ_0,001,721 may participate in the glucose metabolism of OS by activating MAPK pathway. In view of the complexity and network of the pathways, it has not yet been studied whether there are common targets among these pathways to regulate the glycolysis of OS(Fig. 2). A better understanding of the relationship between OS glucose metabolism processes and signaling pathways will help optimize the treatment strategy of OS. In addition, JNK pathway [144], Notch pathway [145], Fas/FasL signaling pathway [146], Hedgehog signaling pathway [147], DNA damage response signaling pathway [148], VEGF signaling pathway [149] and other cell signaling pathways are closely related to the progression of OS. However, the relationship between the above signaling pathways and OS cell glucose metabolism remains to be explored. For example, it has been shown that JNK can regulate the glucose metabolism pathway in glioma mice and the expression of HK2 in glioma mice is significantly upregulated when using JNK inhibitors [150]. In *Drosophila* cells, it was found that, after the Notch signalling pathway was activated, the expression of hexokinase A (hex-A) and ecdysone inducible gene L3 (Impl3, LDHA homolog) was significantly upregulated, similar to the Warburg effect [151]. Similarly, it was found that the Notch pathway activated in mouse bone marrow mesenchymal stem cells reduces glucose consumption and lactate secretion by upregulating NICD2 expression, thereby inhibiting glycolysis pathways [152]. The SHH signalling pathway was found (Fig. 4) to promote glycolysis and breast cancer progression by enhancing the expression of PFKFB3, a key enzyme in the glucose metabolism pathway of breast cancer cells [153]. It can be seen that JNK pathway, Notch signaling pathway, and Hedgehog signaling pathway are related to glucose metabolism pathway, so we speculate that these pathways may also be involved in the glucose metabolism reprogramming of OS cells (Fig. 5).

To sum up, non-coding RNA and small molecular substances participate in the occurrence, progress and metastasis of OS by regulating the expression of key enzymes in the glycolysis process. At the same time, non-coding RNA and small molecule substances can also participate in the regulation of OS glycolysis by affecting the activity of mTOR pathway, hippo pathway, Wnt pathway, NF-κB signaling pathway, MAPK pathway, etc., and ultimately affect the occurrence, progression and metastasis of OS. In addition, Natural extracts (apigenin, caudatin, resveratrol) and chemical compounds (2 -((3-cyanopyridine 2-yl) thio) acetamide, F-AgÅPs, CDDO-NFM, metformin, pramlintide, glutaminase 1 inhibitor) can affect OS progress by targeting the regulation of glucose metabolism.Therefore, the role of reprogramming in the regulation of glucose metabolism in the occurrence and development of OS cannot be ignored, and it is expected to be an effective strategy to treat OS by regulating the glucose metabolism pathway. Previous research has focused on aerobic glycolysis in glucose metabolism, often ignoring the tricarboxylic acid cycle and pentose phosphate pathway. However, the tricarboxylic acid cycle and the pentose phosphate pathway are also involved in the progress of OS, although the specific molecular regulation mechanism is unknown. Therefore, in-depth discussion of the role of tricarboxylic acid cycle, pentose phosphate pathway and its key enzymes in the occurrence and development of OS cannot be ignored. Through in-depth research of the glucose metabolism reprogramming process of OS, more can be known about the mechanism of chemoresistance of OS, which will enable the screening of effective drugs. How to build targeted regulatory drugs to treat OS will also become a new challenge.

authors' contributions

Fangyu An and Weirong Chang wrote the article. Jiayi Song, Jie Zhang, Peng Gao Yujie Wang and Zhipan Xiao drew the figures of the manuscript. Chunlu Yan proposed the the conception or design of the work. All authors edited and check the final manuscript.

Funding

This study was supported by the Gansu Province Higher Education Innovation Fund Project (2022A-072); the Young Doctor Fund Program of Colleges and Universities, Gansu Province (2022QB-091) and the Key Scientific Research Program of“Double first-class”, Gansu Province (GSSYLXM-05).

## CRediT authorship contribution statement

**Fangyu An:** Writing – original draft. **Weirong Chang:** Writing – original draft. **Jiayi Song:** Data curation. **Jie Zhang:** Data curation. **Zhonghong Li:** Data curation. **Peng Gao:** Data curation. **Yujie Wang:** Data curation. **Zhipan Xiao:** Data curation. **Chunlu Yan:** Conceptualization.

## Declaration of competing interest

The authors declare that they have no known competing financial interests or personal relationships that could have appeared to influence the work reported in this paper.
